# Laryngopharyngeal reflux and psychological distress: a vicious cycle worth investigating

**DOI:** 10.1007/s00405-025-09313-z

**Published:** 2025-04-21

**Authors:** Maria Rosaria Barillari, Giulia Maria Giordano, Giuseppe Costa, Edoardo Caporusso, Federica Giumello, Salvatore Tolone, Jerome R. Lechien, Antonino Maniaci, Carlos Maria Chiesa-Estomba, Miguel Mayo Yanez, Andrea Nacci, Armida Mucci, Silvana Galderisi, Luca Bastiani

**Affiliations:** 1 Department of Mental and Physical Health and Preventive Medicine, “L. Vanvitelli” University, Naples, Italy; 2 Laryngology Study Group of the Young-Otolaryngologists of the International Federations of Oto-Rhino-Laryngological Societies (YO-IFOS), Marseille, France; 3 Department of Psychiatry, “L. Vanvitelli” University, Naples, Italy; 4 Division of Phoniatrics and Audiology, “L. Vanvitelli” University Hospital, Nples, Italy; 5 Department of Advanced Medical and Surgical Sciences, University of Campania Luigi Vanvitelli, Naples, Italy; 6 Division of Laryngology and Bronchoesophagology, EpiCURA Hospital, University of Mons, Mons, Belgium; 7 Department of Medicine and Surgery, University of Enna “Kore”, 94100 Enna, Italy; 8 Department of Otorhinolaryngology-Head and Neck Surgery, Hospital Universitario Donostia, San Sebastian, 20003 Spain; 9 Department of Otorhinolaryngology-Head and Neck Surgery, Hospital San Rafael (HSR), Coruña, 15006 A Spain; 10 ENT, Audiology and Phoniatrics Unit, University of Pisa, Pisa, Italy; 11 Epidemiology Section, CNR Institute of Clinical Physiology, Pisa, Italy

**Keywords:** Reflux, Laryngopharyngeal reflux, Stress, Anxiety, Depression, Psychological distress

## Abstract

**Objectives:**

To investigate the correlation between laryngopharyngeal reflux (LPR) and psychological distress in a sample of adult Italian patients.

**Methods:**

LPR was assessed using the Reflux Symptom Index (RSI), Reflux Finding Score (RFS), and 24-hour impedance-pH monitoring. Psychological distress was evaluated with the following clinical tools: the Hospital Anxiety and Depression Scale (HADS), the Hamilton Anxiety Rating Scale (HAM-A), the Hamilton Depression Rating Scale (HAM-D), the Impact of Event Scale-Revised (IES-R), the Insomnia Severity Index (ISI), and the Perceived Stress Scale-10 (PSS-10). Associations between RSI, RFS, and psychological scores were analyzed.

**Results:**

A total of 45 patients with LPR (Study Group, SG) and 29 healthy volunteers (Control Group, CG) were included in the study. Psychological assessments revealed significant differences between the CG and SG, except for the ISI. The HAM-A score was 6.79 ± 6.5 in the CG versus 9.53 ± 5.8 in the SG (*p* = 0.025), with similar results for the HADS (*p* = 0.029). For the HAM-D, mean scores in both groups were below the threshold for mild depressive symptoms, though SG scores were just below the cut-off (CG: 4.86 ± 5.1; SG: 6.89 ± 4.1; *p* = 0.010). The PSS-10 indicated mild to moderate perceived stress, with significantly higher scores in the SG (CG: 13.90 ± 5.5; SG: 21.62 ± 8.1; *p* = 0.000). RSI scores were positively correlated with HAM-D, HADS, and HAM-A scores.

**Conclusions:**

Psychological distress is significantly higher in LPR patients compared to healthy controls. These preliminary findings suggest that psychological factors should be considered in the management of LPR.

**Supplementary Information:**

The online version contains supplementary material available at 10.1007/s00405-025-09313-z.

## Introduction

Laryngopharyngeal reflux (LPR) is a clinical condition caused by the direct and/or indirect effects of gastroduodenal content reflux into the upper aerodigestive tract, leading to morphological and/or neurological changes [1]. Clinical symptoms may include dysphonia, dysphagia, throat pain, globus sensation, excessive throat clearing, postnasal drip, troublesome cough, and coughing after lying down or eating, with or without heartburn and regurgitation [1, 2]. Various risk factors have been associated with the development of LPR, such as tobacco use, alcohol consumption, obesity, primary esophageal dysmotility, and high-fat diets. However, there is increasing interest in psychological distress as a potential triggering or exacerbating factor for reflux.

Several studies have explored the connection between the brain and the gastrointestinal tract, suggesting that psychological distress - particularly in terms of perceived stress, anxiety, and depression - and sleep disorders may act as triggering factors for the onset of gastroesophageal reflux disease (GERD) and LPR [3–7]. Additionally, increased anxiety and stress levels are known to be associated with autonomic nerve dysfunction and related impairments in gastroesophageal motility [8].

Preliminary reports [9, 10] have highlighted psychological distress as a potential cofactor influencing symptom severity in patients with LPR. However, other authors [11, 12] have failed to demonstrate a significant correlation between psychological distress and reflux symptoms. Although most studies in this field have focused on GERD rather than LPR, further research into the relationship between LPR and psychological distress could provide valuable insights for the effective management of symptoms.

Therefore, the current study aimed to: (1) Explore the correlation between LPR and psychological distress in a sample of adult Italian patients diagnosed with LPR via multichannel intraluminal impedance-pH (MII-pH) monitoring, using standardized clinical tools; (2) Correlate LPR clinical data with the presence and severity of psychological distress; (3) Compare psychological distress scores in patients with LPR (Study Group, SG) to those obtained from a group of healthy volunteers (Control Group, CG).

## Materials and methods

### Study design and patient selection

This was a prospective-controlled single-center study. Subjects with a diagnosis of LPR (SG) were prospectively enrolled from the Division of Phoniatrics and Audiology of the “Luigi Vanvitelli” University Hospital, Naples (Italy), from April 2022 to December 2023. Patients met the following inclusion criteria: (1) age range 18–65 years; (2) clinical suspicion of LPR based on a Reflux Symptom Index (RSI) > 13 [13, 14], Reflux Finding Score (RFS) > 7 [15] and positive 24 h MII-pH monitoring [more than 14 reflux episodes recorded at the most proximal site; 16]. Exclusion criteria were: (1) presence of other organic laryngeal disorders requiring medical, rehabilitative or surgical treatment (e.g., tumours or vocal fold paralysis); (2) history of previous medical/surgical treatments, radiotherapy, or voice therapy for head and neck diseases; (3) confirmed neurological or psychiatric illness or ongoing treatment; (4) fibromyalgia syndrome; (5) use of Selective Serotonin Reuptake Inhibitor (SSRIs) in the past 30 days or pharmacological treatment with fluoxetine in the past 3 months; (6) alcohol abuse; (7) history of upper respiratory tract infection or treatment within the past month; (8) active seasonal allergies or asthma; (9) pregnancy; (10) use of PPIs and/or alginates within 30 days prior to laryngological/gastroenterological evaluation; (11) presence of endoscopically documented gastric and/or duodenal ulcer; (12) history of anti-reflux surgery/esophageal surgical procedure; (13) comorbidities such as scleroderma, diabetes mellitus, myopathy or peripheral neuropathy that could affect esophageal sphincters pressure or increase esophageal clearance time; (14) other major concurrent medical condition; (15) inability to provide infromed consent.

A group of healthy volunteers was recruited as the Control Group (CG). Each CG participant underwent a thorough clinical interview to exclude any past or current symptoms of LPR, voice disorders, or significant psychiatric illnesses. The CG was recruited during the same period as the SG and was matched for age, gender, and geographic distribution.

Written informed consent was obtained from all participants in accordance with the ethical principles outlined in the Declaration of Helsinki.

## Clinical and instrumental evaluation

### Evaluation of LPR

LPR was initially suspected based on the administration of the RSI and a videolaryngoscopic examination plus RFS and subsequently confirmed by MII-pH monitoring. Laryngoscopic examinations were performed using a Storz 70° rigid endoscope (KARL STORZ GmbH & Co. KG, Tuttingen, Germany; diameter: 5.6 mm; equipped with an ATMOS Endo-Stroboscope L - ATMOS Medizin Technik GmbH & Co KG, Lenzkirch, Germany). The procedure was managed through Daisy endoscopic software (2014; ver. 3.6.15, Amplifon SPA, Milan, Italy) with a videoendoscopy module (OMVISIA, 2014, ver. 2.0.8 - Amplifon SPA Milan, Italy). For patients unable to tolerate rigid videoendoscopy, a flexible endoscopic examination was performed using a Xion EF-N 3.4 nasopharyngoscope (XION GmbH, Berlin, Germany). All videoendoscopic evaluations were conducted by the same trained laryngologist (G.C.), and the images were anonymized and independently reviewed by two additional laryngologists (M.R.B., A.N.), both experienced in voice disorders and LPR management. The RFS was rated by two independent physicians (M.R.B., A.N.) and, given the subjective interpretation of the scale, inter-rater agreement for the RFS score was calculated. MII-pH monitoring was performed by a trained physician (S.T.) while patients were off acid suppression therapy, in order to confirm reflux episodes. The procedure employed a catheter with impedance electrode pairs located at 3–5, 7–9, and 15–17 cm above the lower esophageal sphincter (LES) and two pH sensors positioned 5 cm and 15 cm above the LES (Sandhill Diversatek, Highlands Ranch, Colorado, USA). Traces were analyzed using Bioview software and manually reviewed by an expert investigator (S.T.). Reflux at the most proximal channel was recorded, and the test was considered positive if more than 14 reflux episodes were detected at the most proximal site.

### Psychological assessment

Both the SG and CG underwent specific screening for psychological distress using validated and standardized questionnaires, as detailed in Table 1. These included: (1) The Hospital Anxiety and Depression Scale (HADS) [17]; (2) the Hamilton Anxiety Rating Scale (HAM-A) [18]; (3) the Hamilton Depression Rating Scale (HAM-D) [19]; (4) the Impact of Event Scale-Revised (IES-R) [20]; (5) The Insomnia Severity Index (ISI) [21]; (6) the Perceived Stress Scale 10 (PSS-10) [22].

The psychological assessment for both groups was conducted by two trained psychiatrists (G.G., E.C.), who were blinded to the participants’ group assignments. These psychiatrists have proven expertise in administering and interpreting the aforementioned clinical tools. For the SG, the psychological evaluation was conducted prior to starting anti-reflux therapy. Additionally, all study participants underwent the Mini-International Neuropsychiatric Interview (M.I.N.I.) [23] to identify any current or previous neuropsychiatric conditions or treatments, as outlined in the exclusion criteria.

**Table 1:** Patient reported outcomes questionnaires

Questionnaire	Domains	Scoring	Cut-off values
HADS	Depression and anxiety (non-physical symptoms)	0–21	0–7 = absent; 8–10 = mild/borderline; > 11 = clinically significant
HAM-A	Anxiety (both psychic and somatic)	0–56	0–7 = absent; 8–14 = mild anxiety; 15–23 = moderate anxiety; > 24 = severe anxiety.
HAM-D (17 items)	Depression	0–53	< 7: absent; 8–17: mild; 18–24: moderate; > 25: severe
ISI	Insomnia	0–28	0–7: absent; 8–14: subclinic; 15–21: moderate; 22–28: severe
IES-R	Acute stress/post traumatic stress disorder	0–88	< 23 = absent 24–32 = moderate impact > 33 = clinically significant
PSS-10	Perceived stress	0–40	0–13: low perceived stress; 14–26: moderate perceived stress; 27–40: high perceived stress

### Statistical analyses

All statistical analyses were completed using SPSS Version 24 (IBM Corp, Armonk, US) and significance was set at *p* < 0.05. Categorical variables were expressed as percentages while continuous variables were expressed as mean ± standard deviation (SD) or interquartile range (IQR). For comparison between categorical and nominal variables, the Pearson Chi-Square and the Fisher’s Exact Test were used, while to compare continuous data, Student’s *t*-test for independent groups was performed. For the comparison of the psychological screening scores, the non-parametric Mann- Whitney U test was performed. A linear relationship between two sets of data has been assessed using Spearman’s rank correlation coefficient.

## Results

Although 92 patients with suspected LPR were initially assessed for eligibility, 12 patients declined to undergo the MII-pH test. Among the remaining 80 patients with positive MII-pH results, 16 did not meet the inclusion/exclusion criteria, and 19 refused to undergo the psychiatric assessment. Additionally, 29 subjects agreed to participate as the Control Group (CG). Thus, the final sample for this study comprised 74 participants: 45 in the SG and 29 in the CG. The sample included 48 females and 26 males, with a mean age of 35.8 ± 13.3 years (SD). The demographics and basic clinical characteristics of both groups are presented in Table 2.

None of the analyzed characteristics - such as sex, age, or tobacco and alcohol consumption (moderate intake) - showed statistically significant differences between the SG and CG (Table 2).

**Fig. 1:**
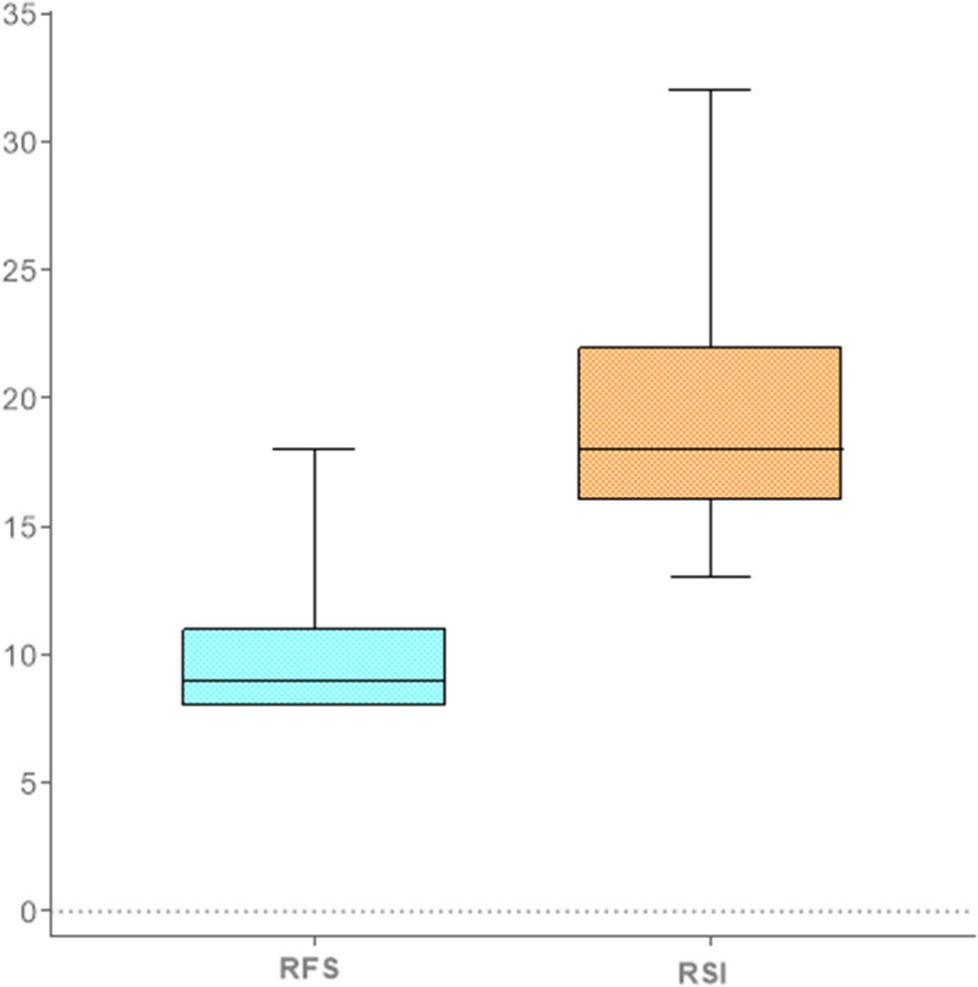
Clinical symptoms and objective signs of LPR in the study group. RFS: Reflux Finding Score; RSI: Reflux Symptom Index

The median RSI total score in the SG was 18 (IQR 16–22) while the median RFS total score was 9 (IQR 8–11), thus suggestive for clinical LPR, as reported in Fig. 1 The overall inter-rater reliability of the RFS, measured as Cohen kappa, was found to be good (kappa = 0.84).

**Table 2:** Sociodemographic and basic clinical characteristics of the two groups

		CG (*n*°29)	SG(*n*°45)	Total (*n*°74)	*p*-value
**Age** (n, mean, SD)	**Years**	29 (33.41; 12.6)	45(37.4;13.6)	74 (35.8;13.3)	0.215
**Gender** (n,%)	**Females**	17 (58.6)	31 (68.9)	48 (64.9)	0.366
	**Males**	12 (41.2)	14 (31.1)	26 (35.1)	
**Tobacco** (n,%)	**Yes**	9 (31.0)	15 (33.39)	24 (32.4)	0.837
	**No**	20 (69.0)	30 (66.7)	50 (67.6)	
**Alcohol** (n,%)	**Yes**	1 (3.4)	5 (11.1)	6 (8.1)	0.238
	**No**	28 (96.6)	40 (88.9)	68 (91.9)	

CG: control group; SG: study group; SD: standard deviation. Differences between the two groups are not significant

**Fig. 2:**
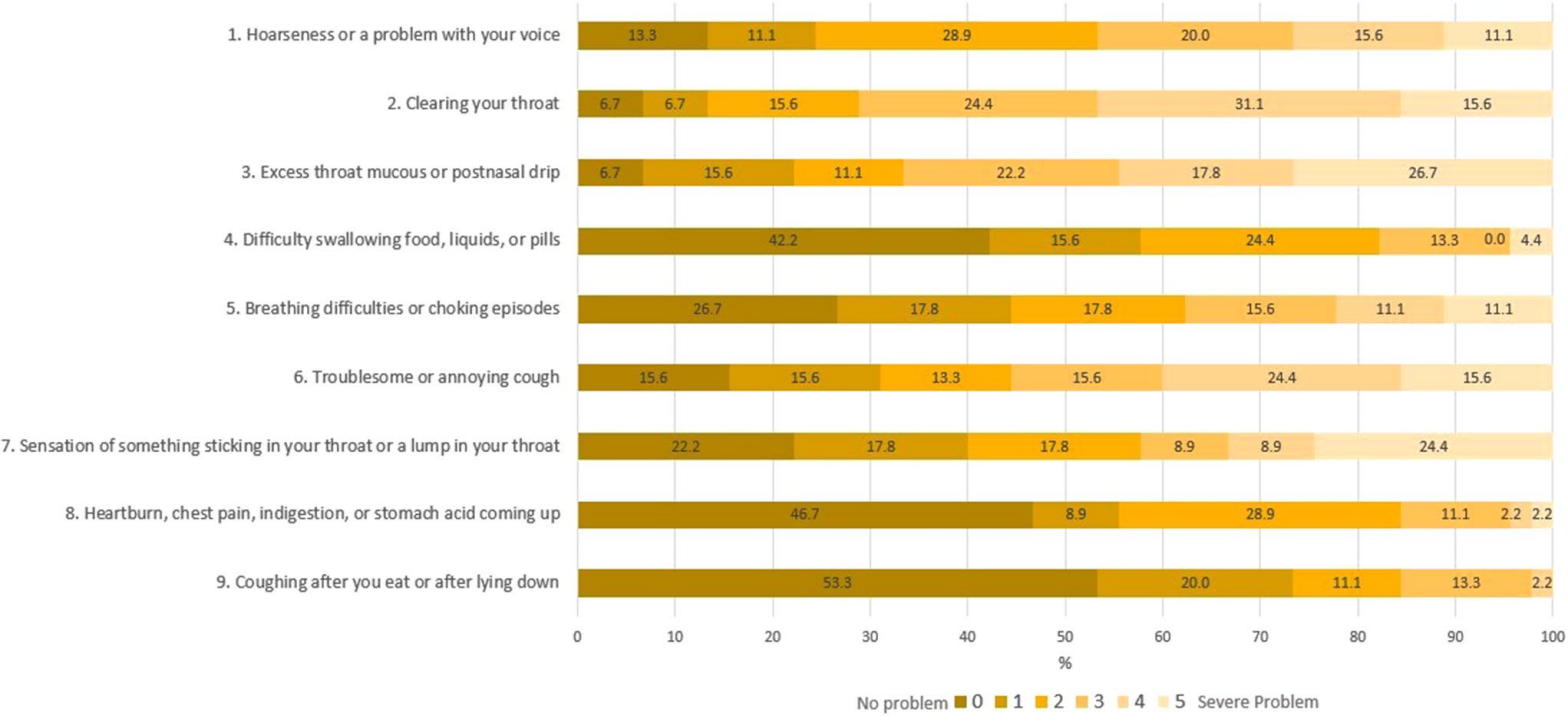
Clinical symptoms distribution in the study group (SG)

**Fig. 3:**
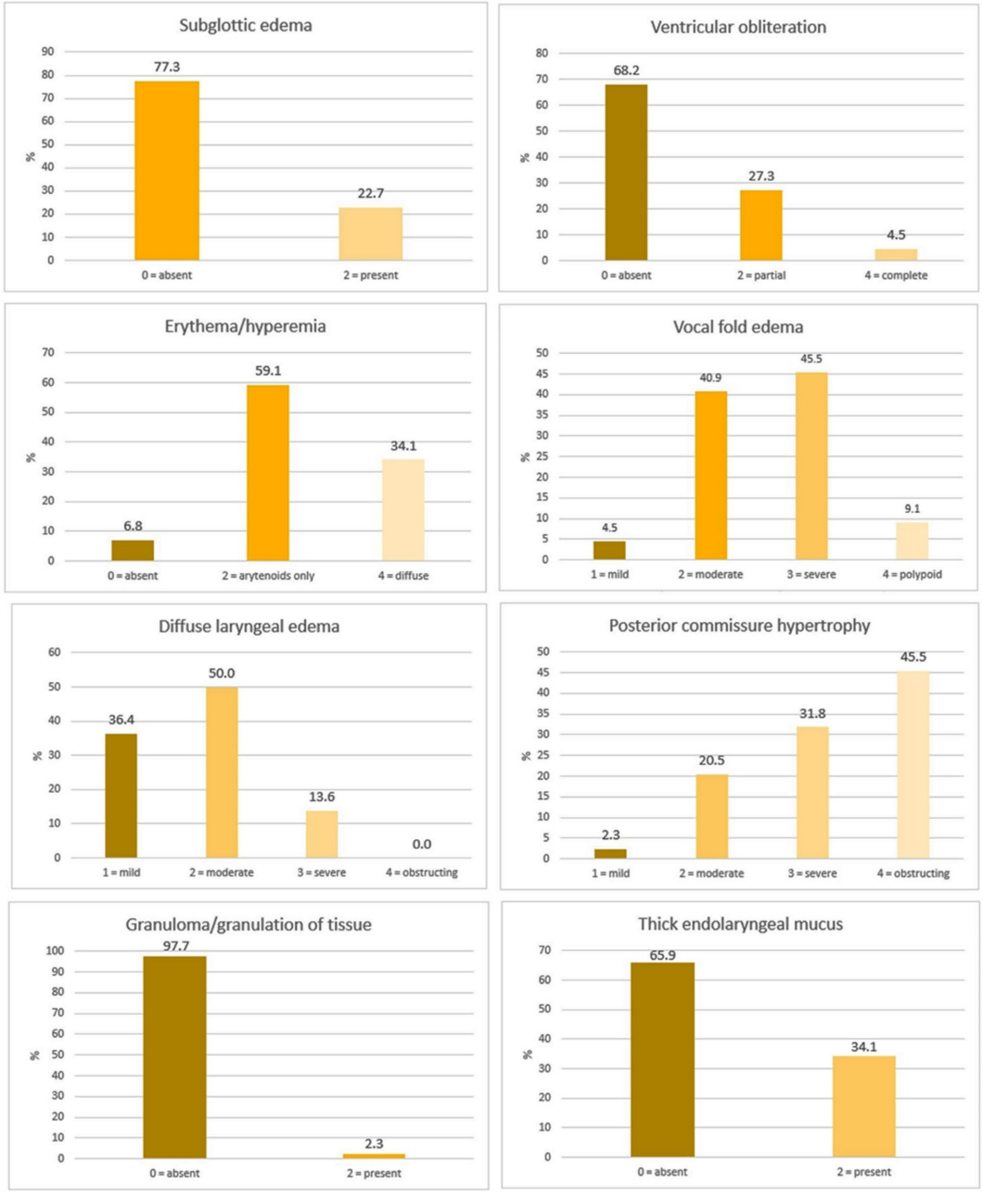
Distribution of the objective laryngeal signs in the SG

The most common clinical symptoms in the SG (Fig. 2) were throat clearing (moderate-to-severe: 46.7%), followed by excess throat mucus (moderate-to-severe: 44.4%) and globus sensation (moderate-to-severe: 40.0%). The main objective laryngeal findings included: arytenoid erythema (59,1%), diffuse laryngeal edema of moderate severity (50%), posterior commissure hypertrophy (severe 31.8%; obstructing 45.5%) and vocal fold edema (moderate 40,9%; severe: 45,5%) as shown in in Fig. 3.

**Fig. 4:**
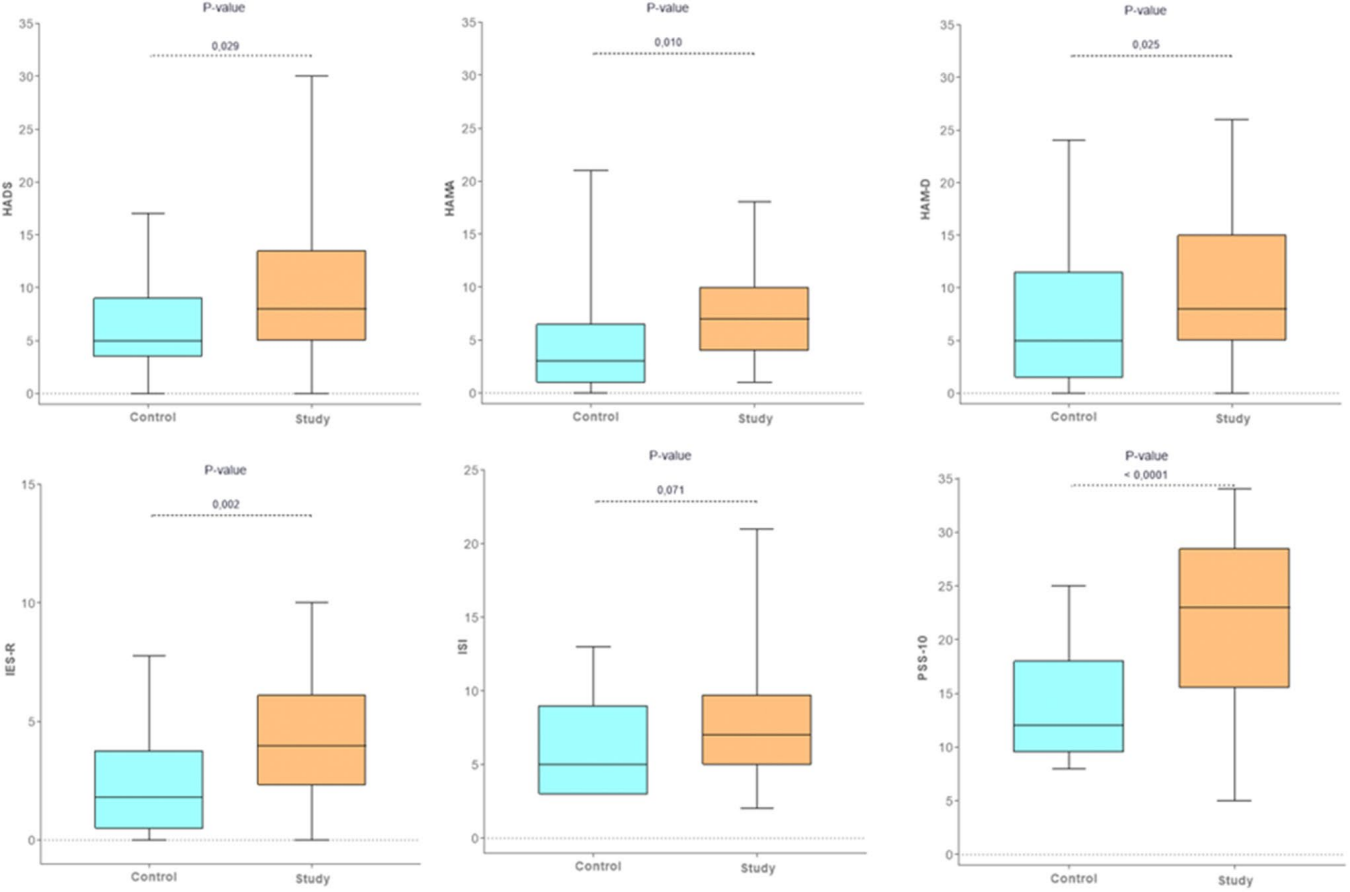
Psychological distress in the CG and SG. HAM-A: the Hamilton Anxiety Rating Scale; HAM-D: the Hamilton Depression Rating Scale; HADS: The Hospital Anxiety and Depression Scale; IES-R: he Impact of Event Scale-Revised; ISI: The Insomnia Severity Index; PSS-10: the Perceived Stress Scale 10

The psychological distress assessment revealed significant differences between the CG and the SG across all clinical tools, except for the ISI, which did not show statistical significance (Table 3; Fig. 4). Specifically, the mean scores for the HAM-A, a psychometric tool for assessing anxiety, were 6.79 ± 6.5 (SD) in the CG and 9.53 ± 5.8 (SD) in the SG (*p* = 0.025). According to *Matza et al.* [18], a HAM-A score < 7 indicates no anxiety, while scores between 8 and 14 suggest mild anxiety. Consequently, patients in the SG not only exhibited significantly higher scores compared to the CG but also demonstrated a greater tendency toward anxiety disorders, albeit at a mild level. Similar results were observed for the HADS, which evaluates symptoms of both anxiety and depression. Scores in the SG (9.69 ± 7.7, SD) were significantly higher than those in the CG (6.21 ± 4.6, SD) (*p* = 0.029), indicating a borderline anxious-depressive syndrome (scores 8–10). For the HAM-D, mean scores in both groups were below the threshold for mild depressive symptoms (< 7). However, the SG’s scores (6.89 ± 4.1, SD) were just below this cutoff, showing a significant difference compared to the CG (*p* = 0.01). No significant differences were observed for the ISI (CG: 6.14 ± 3.2, SD; SG: 8.0 ± 4.5, SD). Nevertheless, the SG’s mean score fell within the range of mild discomfort compatible with subclinical insomnia (scores 8–14), whereas the CG’s mean score indicated no clinically significant insomnia (scores 0–7). Scores on the IES-R also differed significantly between the SG and CG, although both groups remained well below the threshold for post-traumatic stress disorder.

Finally, PSS-10 scores indicated mild to moderate perceived stress levels in our sample. Patients in the SG demonstrated significantly higher scores, indicative of moderate stress, compared to the CG, which showed mild stress.

**Table 3:** Psychological assessment in the two groups

	CG (*n*°29)	SG (*n*°45)	*p*-value
	Mean, SD, Median, IQR		
HAM-A	6.79 (6.5); 5: 1.5–11	9.53 (5.8); 8: 5–15	0.025*
HAM-D	4.86 (5.1); 3: 1-6.5	6.89 (4.1); 7: 4–10	0.010*
HADS	6.21 (4.6); 5: 3.5-9	9.69 (7.7); 8: 5-13.5	0.029*
IES-R	2.19 (2.1); 1.8: 0.5–3.8	4.30 (2.7); 4: 2.3–6.1	0.002*
ISI	6.14 (3.2); 5: 3–9	8.0 (4.5); 7: 5-9.8	0.071
PSS-10	13.90 (5.5); 12: 9.5–18	21.62 (8.1); 23: 15.5–28.5	0.000*

CG: control group; SG: study group; SD: standard deviation; HAM-A: the Hamilton anxiety rating scale; HAM-D: the Hamilton depression rating scale; HADS: the hospital anxiety and depression scale; IES-R: he impact of event scale-Revised; ISI: the insomnia severity index; PSS-10: the perceived stress scale 10. p-value*: significant

**Fig. 5:**
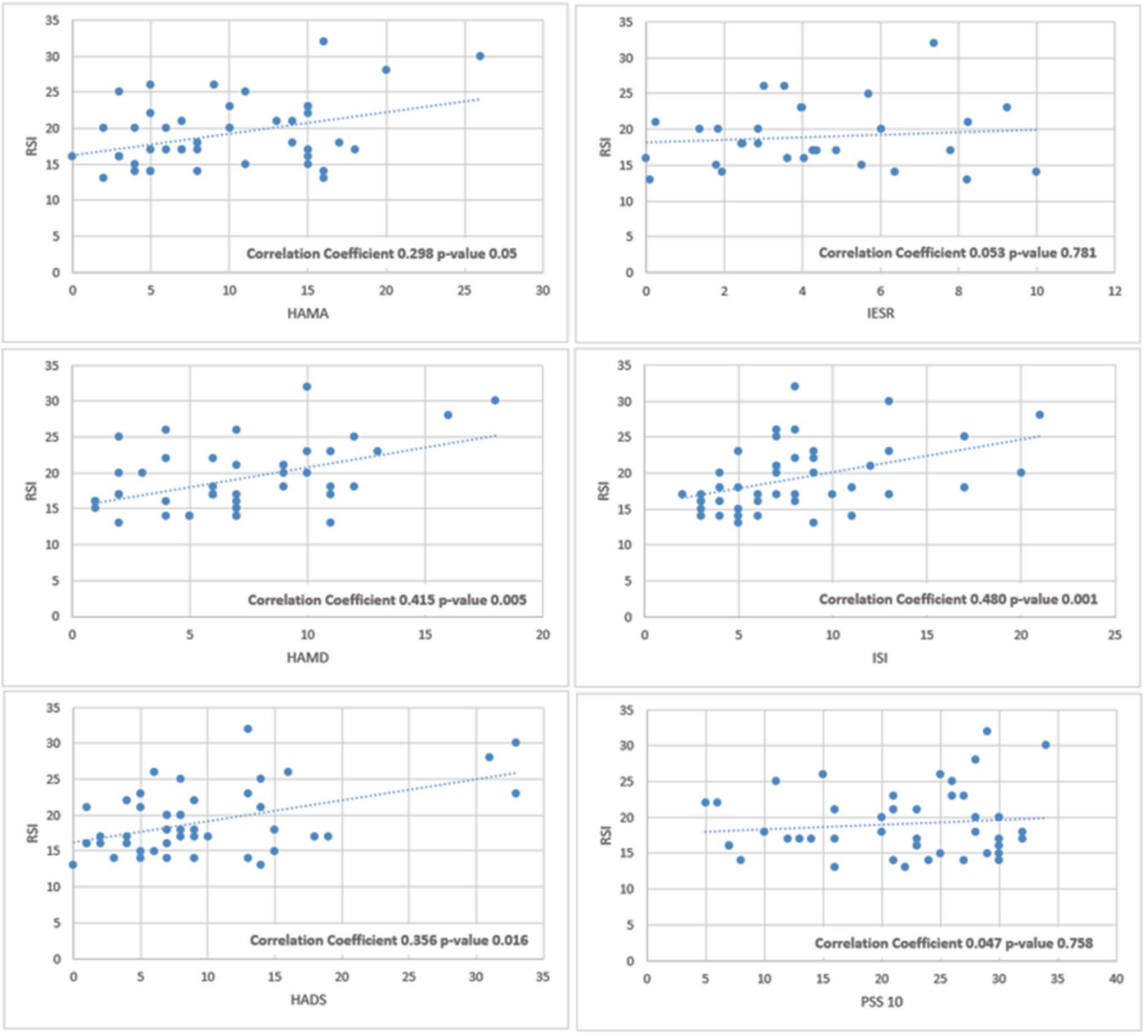
Correlation between the severity of clinical symptoms of reflux (RSI) and psychological distress

The next objective was to correlate the clinical symptoms reported by patients in the SG (RSI) with the results of each psychological assessment tool (Fig. 5). The RSI demonstrated positive correlations with most of the psychological questionnaires used. The strongest correlations were observed between RSI and ISI (Spearman’s rank correlation coefficient 0.480), RSI and HAM-D (Spearman’s rank correlation coefficient 0.415), RSI and HADS (Spearman’s rank correlation coefficient 0.356), and, although lower, between RSI and HAM-A (Spearman’s rank correlation coefficient 0.298). No correlations were found between the RSI and the IES-R or PSS10. In contrast, when analyzing the correlation between objective laryngeal signs (RFS) and psychological questionnaires, no significant associations were evident.

Based on these findings, LPR patients with higher RSI scores are more likely to exhibit subclinical insomnia, mild anxiety, and a borderline anxious-depressive syndrome. However, while stress levels scores (PSS-10) were significantly higher in the SG, they did not correlate with the severity of the RSI.

## Discussion

Psychological distress may contribute to the onset or exacerbation of LPR-related symptoms, although the correlation between mental health and reflux symptoms has not yet been fully clarified. A sort of vicious cycle has been described, wherein psychological distress and emotional tension negatively impact gastrointestinal function, while the severity of a gastrointestinal disorder can, in turn, affect a person’s psychological and emotional well-being. Our preliminary data indicate a greater tendency toward stress, anxiety, and depressive symptoms in LPR patients. Although these results are consistent with data previously reported by other authors [9, 10, 24], this tendency does not necessarily indicate the presence of full-blown psychiatric disorders.

*Cheung et al.* [9]. observed that LPR patients had higher anxiety scores compared to controls, negatively impacting psychological status, social functioning, and quality of life. *Suipsinkiene et al.* [10] reported significant psychological distress, particularly anxiety, in LPR patients compared to healthy controls, noting that quality-of-life impairment was more strongly associated with symptoms than with laryngeal findings. In both studies, LPR patients were clinically defined without the use of pH monitoring.

*Gong et al.* [24] demonstrated that GERD patients with LPR symptoms experienced more depression and anxiety than those without LPR symptoms, while *Laohasiriwong et al.* [25]. found that GERD patients with LPR symptoms were more likely to experience sleep disturbances than those without such symptoms. Additionally, multiple overlapping psychological distress factors were more prevalent in GERD patients with LPR symptoms than in those with GERD alone. Similarly, *Wong et al.* [26] indicated that GERD patients with LPR symptoms exhibited higher levels of depression and sleep disturbances compared to those with GERD alone. However, regarding anxiety, the difference between these groups did not reach statistical significance.

Although the studies by *Shin et al.* and *Mesallam et al.* [11, 12] did not find a correlation between anxiety and LPR, our findings support this association. According to our results, mild anxiety is significantly more prevalent in the SG group compared to controls, often exacerbating symptoms commonly associated with LPR, such as dysphonia, throat clearing, and globus sensation [27]. In our opinion, and in line with the literature on the relationship between anxiety and voice disorders [28], the presence of anxiety and/or somatization could worsen secondary muscle tension dysphonia (MTD) to which patients with LPR are predisposed. The etiological classification of MTD clearly shows that psychological and/or personality factors play a crucial role in its development, with several models linking vocal disorders and psychosocial distress. These include: (1) the ‘disability model,’ where a physical vocal disorder leads to psychosocial issues; (2) the ‘vulnerability model,’ where a psychosocial disorder causes physical vocal problems; or (3) a combination of both. A vicious circle may be formed, where each condition, if not identified and treated early, worsens the other, exacerbating the clinical picture [28].

Regarding the pathophysiological mechanisms of LPR, studies show that the vocal cord cover undergoes various microstructural changes due to acidic pepsin. These include downregulation of mucin genes, chronic mucus dehydration, endolaryngeal sticky mucus, dryness of the superficial layer of the lamina propria, and increased viscosity [1]. These alterations lead to a reduction in the amplitude of the free edge of the vocal folds and greater vocal effort. As a result, hyperfunctional behaviors of the thyroarytenoid muscle develop due to the inflammatory reaction on the surface, contributing to the onset of MTD. This condition is driven by increased muscular tension in the larynx and neck. The increased phonatory muscle tension affects the paralaryngeal and suprahyoid muscles, causing an altered position of the larynx within the neck and resulting in incomplete glottic closure in the posterior third of the glottal plane. These changes can influence the stability and contractility of the upper esophageal sphincter (UES), whose function is closely related to the position of the larynx. It is optimal when the larynx is in a neutral position but becomes impaired when it deviates from this alignment. This condition is further exacerbated by the vicious cycle between anxiety, stress, and MTD. Consequently, there may be an additive effect between anxiety and LPR in the development of secondary MTD.

*Wook Kang et al.* [29] did not find significant levels of anxiety in LPR patients but reported greater somatization (somatic anxiety) compared to the control group. They also found a significant correlation between RSI and somatic anxiety. Somatization, the physical manifestation of anxiety, refers to the phenomenon in which patients experience and express their emotions or psychological distress through physical symptoms. Although somatization is included as an item in the HAM-A, it was not specifically evaluated in our study and should be considered for future research.

The greatest significance (*p* < 0.0001) between CG and SG was observed in perceived stress (PSS-10), suggesting a relationship between stress and LPR symptoms. While this association is not new, it has been rarely studied and is often underestimated in the clinical and rehabilitative approach to LPR patients. Some common symptoms of LPR, such as globus sensation and oropharyngeal dysphagia, may be directly associated with stress and anxiety or exacerbated by psychological factors [30, 31].

Patients with higher stress and anxiety scores may exhibit overall autonomic nervous system dysfunction, particularly an imbalance in the sympathetic–vagal axis, with increased sympathetic activity and vagal nerve dysfunction [32, 33]. The heightened sympathetic activity related to stress and anxiety leads to abnormal regulation of gastric peristalsis, resulting in transient relaxation of the esophageal sphincter. This, in turn, increases distal and proximal reflux episodes, causing significant enzyme deposition in the mucosa of the upper aerodigestive tract [34].

*Wang et al.* observed that LPR severity was significantly correlated with autonomic nerve dysfunction [35] and that some LPR patients exhibited signs of mild anxiety and depression; similar results were reported by *Huang et al.* [36]. A further study by *Hu et al.* [37] confirmed that patients with anxiety and depression experienced marked autonomic nerve dysfunction, which significantly improved after their anxiety and depression were treated.

A rat model study conducted by *Farre et al.* [38] found that acute stress alone can potentiate the effect of acid-pepsin on the esophageal mucosa by increasing its permeability. Although similar mechanisms have not been specifically described in the laryngopharyngeal tract, it is reasonable to hypothesize that stress plays a role in the heightened perception of LPR symptoms.

Furthermore, preliminary studies suggest that stressed LPR patients may exhibit a lower therapeutic response compared to those who are not or are less stressed [31, 34, 39].

The scores related to insomnia (ISI) did not show significant differences between the CG and SG, contrary to findings reported by *Laohasiriwong et al.* and most GERD studies. *Kurin et al.* [40] noted a close relationship between GERD and sleep disturbances, though the exact nature of this relationship remains unclear. A bidirectional relationship has been proposed, wherein GERD can lead to sleep deficiency, and sleep deficiency, in turn, exacerbates GERD, creating a vicious cycle [41]. However, this correlation may be less pronounced or absent in LPR patients compared to GERD patients, likely due to the physiopathological and clinical characteristics of LPR. Unlike GERD, LPR predominantly manifests as daytime symptoms occurring in an upright position, which interferes less with sleep quality.

In our sample, insomnia was not frequent in either the CG or SG, and when present, it was generally mild or subclinical. However, it was correlated with the severity of RSI.

## Limitations

This study has some important limitations.

First, the number of enrolled patients is relatively small, and the results should therefore be considered preliminary. The limited sample size was partly due to the adoption of very selective inclusion and exclusion criteria, which led to the exclusion of a certain number of patients from the final analysis. Furthermore, 19 patients who met the inclusion criteria declined psychological assessment. It is worth noting that patients may fear being judged based on their responses to psychological evaluations, which could have influenced the final data. Overcoming the stigma associated with psychological distress remains challenging, and assisting patients in this regard could be key to developing integrated treatment approaches. In our clinical protocol, psychological assessments were always conducted with the presence and assistance of trained psychiatrists. This may have inhibited some patients, potentially affecting the honesty of their responses and leading to lower scores on tests, particularly those assessing depression and post-traumatic stress disorder.

Second, we used the RSI and RFS scales to assess symptoms and findings associated with LPR. While these scores are widely referenced in the literature, the use of newer validated clinical tools for diagnosing LPR—tools that also account for extra-laryngeal symptoms and findings—might be recommended in future studies.

Third, all study participants were seen at a single academic voice clinic. Conducting international multicenter studies on this topic would help rule out any potential influence of environmental factors specific to the geographic region on psychological well-being.

Fourth, the diagnosis of LPR was made using event-number criteria based on prior research comparing the traditional MII-pH catheter configuration (which we used) to the newer “LPR” type MII-pH catheter configuration, also known as hypopharyngeal-esophageal multichannel intraluminal impedance with dual pH testing (HEMII-pH). While the newer HEMII-pH catheters offer a more precise approach, we opted to use the current MII-pH catheter data to extrapolate the number of pharyngeal events. This approach was deemed more appropriate than using acid-only/GERD criteria, which might have overlooked patients with true LPR.

Finally, no follow-up psychological assessments were planned for the SG after two months of treatment. Such follow-up assessments could be valuable for evaluating whether patients with higher scores for psychological distress showed improvement after targeted dietary and pharmacological treatments, and for correlating these improvements with the RSI total scores post-therapy. In cases of minimal or no improvement, integrated treatments addressing anxiety and stress management might be recommended.

## Conclusions

Our preliminary report identified a high prevalence of psychological distress, particularly anxiety and moderate perceived stress, among patients diagnosed with LPR who had no prior specific psychiatric diagnosis. For this reason, we believe these symptoms should be carefully considered in the diagnostic, therapeutic, and follow-up processes for LPR patients. Understanding the relationship between mental health and LPR-related symptoms is crucial, as co-occurring mental health issues may influence key patient-specific factors, including readiness for change, treatment adherence, outcomes, and patient satisfaction. These results highlight the importance of considering LPR from a more holistic perspective. Laryngologists and phoniatricians should be aware of the potential relationship between LPR symptoms and psychological distress, as, in specific cases, psychological assessment and support may be strongly recommended to enhance therapeutic outcomes.

## Electronic supplementary material

Below is the link to the electronic supplementary material.

Supplementary Material 1
