# Supplementary material for: Laryngopharyngeal reflux and psychological distress: a vicious cycle worth investigating
Source: Eur Arch Otorhinolaryngol. 2025 Apr 21;282(6):3103–13. doi: 10.1007/s00405-025-09313-z (PMC12122629; doi:10.1007/s00405-025-09313-z)
Supplement: Supplementary file 1 — Supplementary Material 1 [file 405_2025_9313_MOESM1_ESM.docx]

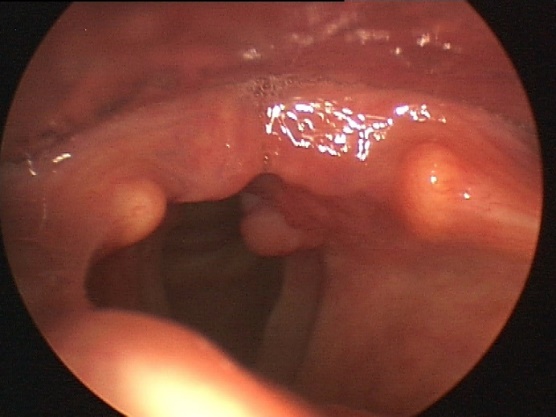


a


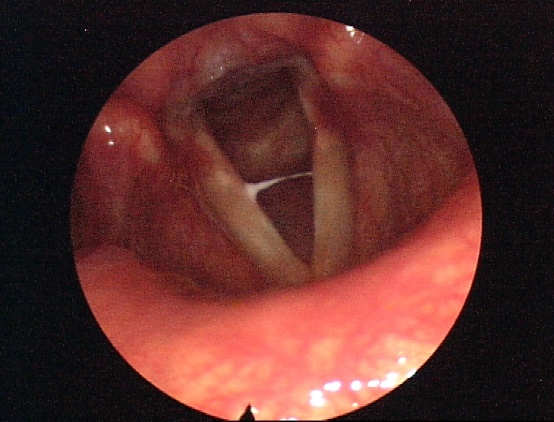


b

Fig. 6. **Videolaryngoscopic images of LPR patients (RFS > 7).**
**Fig. 6 (a):** Moderate vocal fold edema, vocal process granuloma, posterior commissure hypertrophy and diffuse laryngeal hyperemia
**Fig. 6 (b):** Thick endolaryngeal mucus, mild vocal fold edema, posterior commissure hypertrophy and diffuse laryngeal hyperemia
